# Risk Factors for Spinal Cord Injury during Endovascular Repair of Thoracoabdominal Aneurysm: Review of the Literature and Proposal of a Prognostic Score

**DOI:** 10.3390/jcm12247520

**Published:** 2023-12-05

**Authors:** Laurent Brisard, Salma El Batti, Ottavia Borghese, Blandine Maurel

**Affiliations:** 1 Department of Anesthesiology and Critical Care, Laënnec Hospital, University Hospital of Nantes, F-44000 Nantes, France; laurent.brisard@chu-nantes.fr; 2 Department of Vascular and Endovascular Surgery, Hôpital Européen Georges Pompidou—Hôpitaux de Paris, Université de Paris Cité, F-75015 Paris, France; salma.el-batti@aphp.fr; 3 Department of Cardiac and Vascular Surgery, L’Institut du Thorax, Nantes University Hospital, F-44093 Nantes, France; ottaviaborghese@gmail.com; 4 Inserm UMR 1087/CNRS UMR 6291, L’Institut du Thorax, Université de Nantes, F-44000 Nantes, France

**Keywords:** endovascular aortic repair, spinal cord injury, spinal cord ischemia, paraplegia, cerebrospinal fluid drainage, spinal cord preservation

## Abstract

Despite recent improvements, spinal cord ischemia remains the most feared and dramatic complication following extensive aortic repair. Although endovascular procedures are associated with a lower risk compared with open procedures, this risk is still significant and must be considered. A combined medical and surgical approach may help to optimize the tolerance of the spinal cord to ischemia. The aim of this review is to describe the underlying mechanism involved in spinal cord injury during extensive endovascular aortic repair, to describe the different techniques used to improve spinal cord tolerance to ischemia—including the prophylactic or curative use of spinal drainage—and to propose our algorithm for spinal cord protection and the rational use of spinal drainage.

## 1. Introduction

The estimated worldwide incidence of spinal cord injuries is between 40 and 80 new cases per million inhabitants per year. This means that between 250,000 and 500,000 people suffer a spinal cord injury every year [1]. Traumatic spinal cord injury is still the most common type of mechanism. Nontraumatic causes are dominated by the ischemic mechanism, which is mainly related to diseases of the aorta. Spinal cord ischemia (SCI) in aortic repair is an old topic of investigation and discussion, first mentioned by Stenonis and Swammerdam in 1667. As early as 1956, Herbert D. Adams emphasized in an essay that the spinal cord is the only organ in which the effects of aortic occlusion are unexplained and that the exact mechanism is unknown [2].

The treatment of complex thoracoabdominal aneurysms has undergone a minimally invasive revolution in the last 20 years. Endovascular repair is now the method of choice for the majority of extensive and complex aortic aneurysms that require extensive incisions and prolonged aortic clamping, as opposed to open repair, which is associated with significant morbimortality.

However, the further development of these complex endovascular techniques is only foreseeable if the adverse events are under control. SCI remains the most feared complication by patients and physicians, as this adverse event completely reverses the benefits of preventive treatment due to its catastrophic consequences: physical and psychological consequences, health costs, and severe disability. In addition, it has a significant impact on the long-term mortality of patients, especially those with permanent damage. The most recent analysis of 1681 patients showed a 1-year survival rate of 90.8% in patients who did not develop SCI versus 73.9% in patients who developed SCI (84.8% in those who developed paraparesis and 66.2% in those who developed permanent deficits) [3]. Regardless of the type of SCI (i.e., temporary or permanent), patients who underwent SCI after TEVAR had a significantly lower 1-year survival rate than patients who did not undergo SCI (65% vs. 87%) [4]. Permanent SCI was associated with a worse 1-year survival rate than transient SCI (54% vs. 80%).

Although endovascular repair has reduced the risk of SCI compared with the open strategy, this risk is still high, ranging from 4% to 50% in the literature [5,6] and 14% in large recent multicenter studies [7]. The SCI rate is lower in high-volume centers and appears to be decreasing in recent years following the use of rigorous multimodality spinal cord preservation strategies [8]. The explanations for this lower rate compared with open surgery are multiple: no aortic clamping, better hemodynamic stability, lower incidence of anemia, faster awakening and recovery.

The mechanisms of occurrence, the risk factors, and the various medical and surgical strategies to prevent SCI need to be fully understood and applied by a multidisciplinary aortic team offering extensive aortic repair. Risk assessment for SCI is a key component for prevention to apply a range of additional preventive medical and surgical measures that are not limited to cerebrospinal fluid drains (CSFDs).

This manuscript reports a preventive multimodal approach to prevent SCI in extensive endovascular repair as described in the literature and applied in our center.

## 2. Pathogenesis of SCI after Extensive Aortic Endovascular Repair

**Figure 1:**
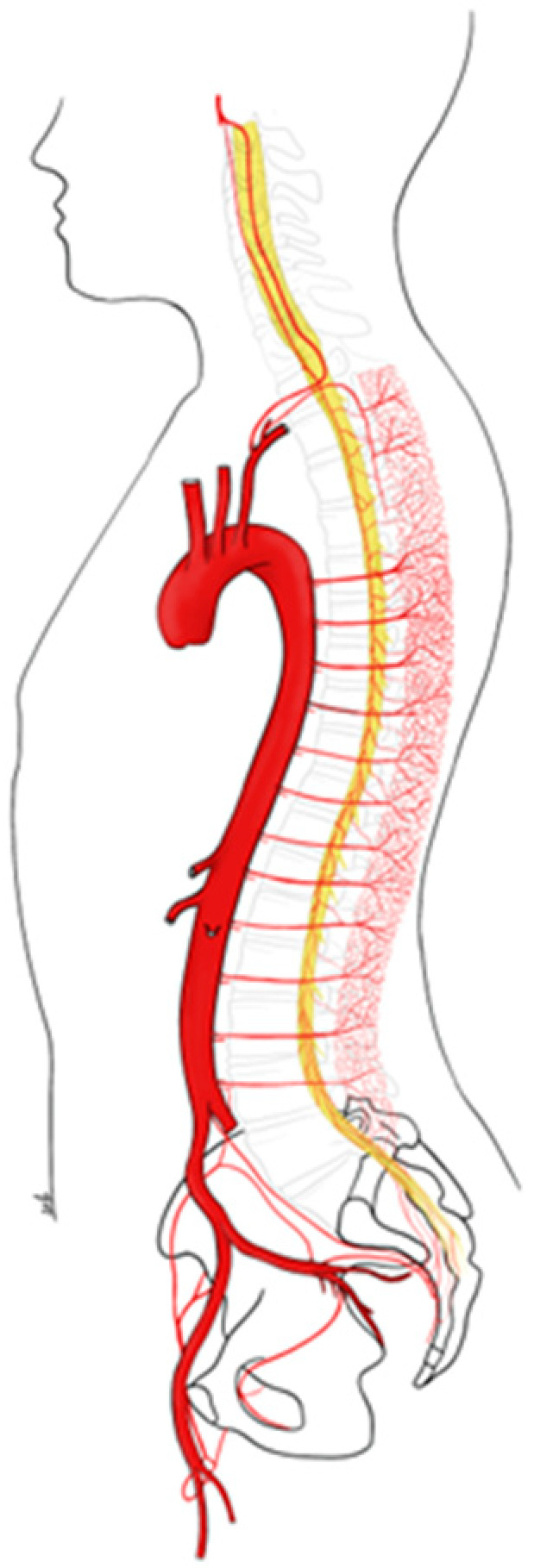
Blood supply to the spinal cord.

The blood supply to the spinal cord appears to be more complex than the blood supply to any other system [9,10]. The spinal cord is supplied with blood by an extensive vascular network that extends into the paraspinous muscles and is connected to the subclavian, hypogastric, and internal thoracic arteries [11]. The intercostal and the lumbar arteries give off branches to the spinal arteries, namely the radiculomedullary arteries, which are connected by longitudinal branches and form a dense collateral network. Finally, there is an anterior spinal artery (ASA) and two posterior spinal arteries (PSAs) that run longitudinally along the spinal cord and originate from the vertebral arteries (Figure 1). The largest of the radiculomedullary arteries, the artery of Adamkiewicz, arises most commonly between T9 and T12 on the left and is also a major branch of the ASA [12]. Although in the past it was considered the main vessel supplying the spinal cord, this has been largely questioned, especially since the development of the endovascular era. Several attempts to preserve direct flow into the artery of Adamkiewicz have failed to demonstrate significant benefit.

Thoracic endovascular aortic repair (TEVAR) permanently excludes many of the segmental arteries that are occluded by the covered stent, which may have longer-lasting effects on blood flow in the spinal cord. Remodeling of the collateral network is thought to play an important role in maintaining blood flow in the spinal cord by altering the distribution of blood flow in the intraspinal and paraspinal network after exclusion of the segmental arteries. The restoration of blood flow from the paraspinous to the intraspinous network helps to maintain the viability and function of the spinal cord.

**Figure 2:**
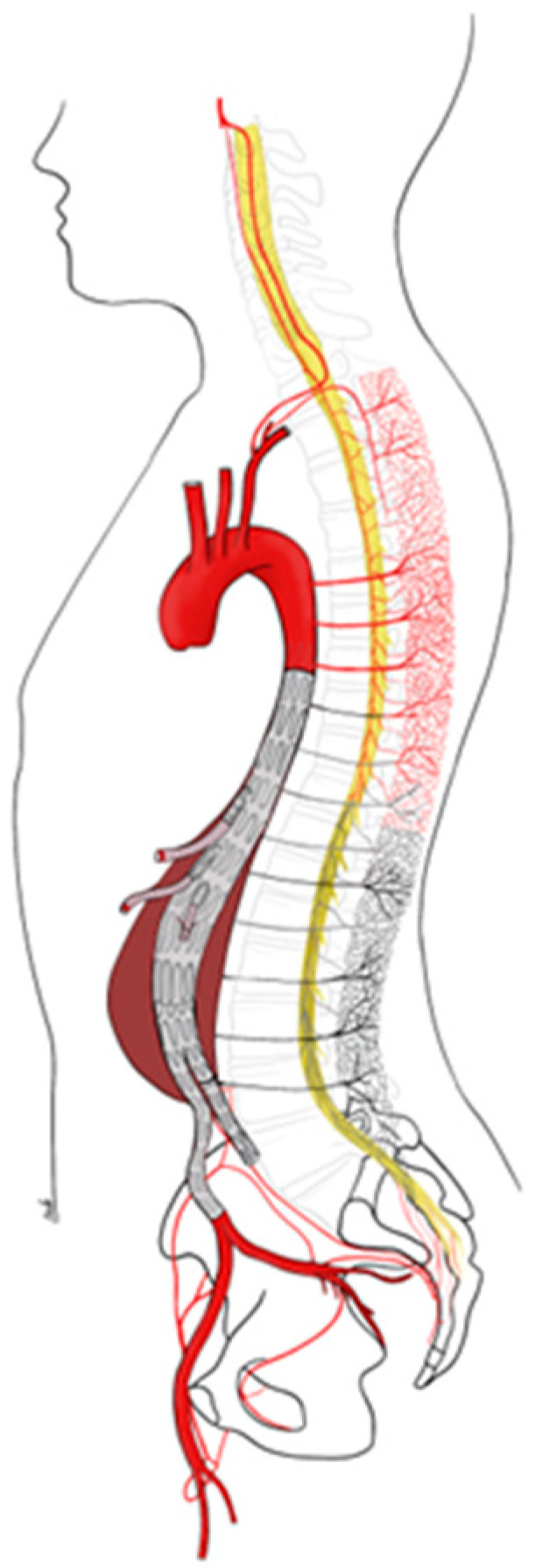
Spinal cord injury following extensive coverage of lumbar and intercostal arteries and pre-existing occlusion of branches of the internal iliac artery.

If these compensatory mechanisms are not sufficient or if there is extensive occlusion (due to thrombus formation or embolism) in the intraspinal or paraspinal network, SCI may occur (Figure 2). The occurrence and severity of damage after TEVAR depends on the ability of the collateral network to supply the marginally vascularized area in the spinal cord.

**Figure 3:**
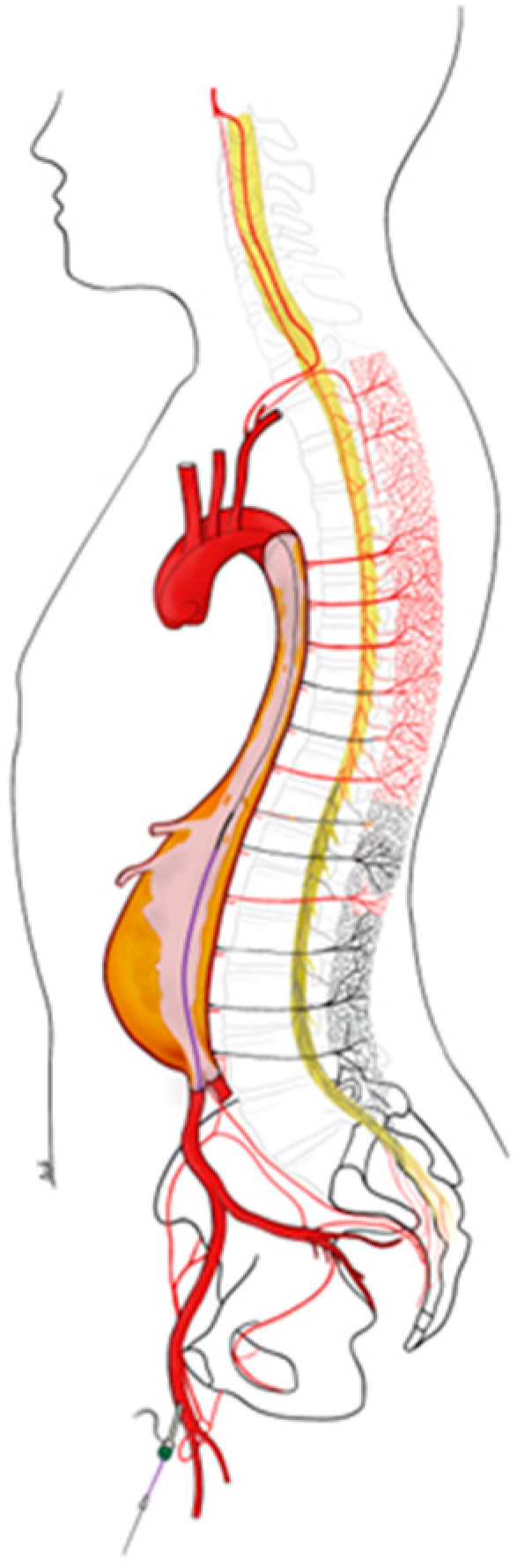
Spinal cord injury due to thrombus embolism and pre-existing occlusion of the lumbar and intercostal arteries due to the shaggy aorta.

Finally, an important factor that can lead to or exacerbate SCI is embolism and thrombosis of supply branches (Figure 3). This appears to be particularly the case in endovascular interventions, where manipulation of the guidewires in the diseased aorta and placement of stents can lead to dislocation of microemboli in the radiculomedullary arteries [13].

In this context, the maximum symptoms of SCI occur in most patients within 12 to 72 h [14].

## 3. Risk Factors for Paraplegia

The pathogenesis leading to SCI is complex and multifactorial. Numerous reports have documented various predictors of SCI following extensive TEVAR, as several mechanisms may be involved in the neural tissue responses to anoxia, most notably a combination of reduced blood flow to the spinal cord due to coverage of its collateral network, hemodynamic instability, and infarction due to an embolic mechanism. Individual comorbidities of the patients may also play a role. This chapter details the patient’s medical and anatomic conditions, surgical strategies, and hemodynamic considerations associated with an increased risk of SCI.

### 3.1. Patient-Related Factors

- Patient condition

In 1681 patients who underwent complex endovascular aortic repair between 2005 and 2020 as part of the US Aortic Research Consortium, age (age ≥70 years (odd ratio (OR) 1.64; 95% confidence interval (CI) 1.63–1.64; *p* = 0.029)) and a history of peripheral vascular disease (OR 1.65; 95% CI 1.64–1.65; *p* = 0.034) were significantly associated with SCI [3]. Impaired preoperative renal function (glomerular filtration rate < 60 mL/min/1.73 m^2^) is also described as a risk factor (OR 2.43; 95% CI 1.18–4.99; *p* = 0.016) among 243 patients [15].

- Prior history of aortic repair

Several single center studies suggest that prior open infrarenal aortic repair is associated with a higher risk of SCI in the case of proximal thoracic (TEVAR) or thoracoabdominal (TAA) endovascular repair. However, a retrospective review of the Vascular Quality Initiative database found a comparable rate of SCI in 9506 patients treated for TEVAR or TAA and with or without prior repair [16]. The role of prior aortic repair is still under controversy.

- Atherosclerosis

Cumulative cardiovascular risk factors and associated diseases (chronic obstructive pulmonary disease (COPD), obesity, chronic renal insufficiency) have been postulated as markers of widespread peripheral atherosclerotic disease, suggesting that patients may have a compromised collateral network of blood supply to the spinal cord preoperatively. In the end, patients may have a shaggy aorta with a high risk of atheroembolization triggered by intravascular manipulations during surgery. In this case, the Shaggy Aorta Scoring System is a useful method for predicting postoperative embolic complications after TEVAR [17].

- Patient anatomical factors

In the same retrospective review of the Vascular Quality Initiative database of 9506 patients who had undergone extensive endovascular repair, multivariate regression revealed that aortic dissection was an independent factor for postoperative SCI (OR 1.65; 95% CI 1.26–2.16; *p* < 0.001). However, the main parameter associated with SCI is the extent of the aneurysm and consequently the length of aortic coverage [3,18]. In addition, preoperative occlusion of one or both hypogastric or subclavian arteries contributes to reducing alternative inflow routes to the spinal collateral network. Occlusion of a single collateral bed has long been associated with an increased risk of immediate SCI and poor recovery [8]. Preventive revascularization of the left subclavian artery is recommended (level B, class IIa) to reduce the risk of neurological complications such as stroke and SCI [19].

### 3.2. Procedure-Related Factors

- Urgent case

Urgent repairs are more likely to lead to hemodynamic instability if the collateral arterial network is still underdeveloped. In fact, in the large DeBakey Medical Center cohort, emergency repairs were found to more than double the risk of SCI (RRR, 2.31; *p* = 0.002) [20]. An urgent repair in the acute phase of an aortic syndrome appears to be the most dangerous, and its postponement after the 15th day should always be considered when feasible.

- Length of aortic coverage

A large extent of aortic coverage is significantly associated with an increased risk of paraplegia, as shown in a recent large analysis by the US Aortic Consortium (*n* = 1681 patients; OR 4.79; 95% CI 4.77–4.81; *p* < 0.001) [3] and a multicenter Italian cohort treated for thoracoabdominal aneurysms of Crawford extents I–III (*n* = 351 patients; OR 20.90; 95% CI 2.69–162.57; *p* < 0.004) [18]. Feezor et al. concluded that the risk of SCI is increased by 30% for every 2 cm of additional thoracic aortic coverage [21]. Similarly, Bisdas et al. concluded that each percent of aortic coverage above the superior mesenteric artery leads to a 1.03-fold increase in the risk of SCI [22].

- Hypotension and hemodynamic instability

Prolonged hypotension or hemodynamic instability has been shown to contribute to the risk of immediate or delayed SCI [4,20,23,24]. This risk exists at least in the first few weeks after extensive TEVAR. In a large report that focused on the results of 1114 open type II repairs, SCI occurred in 13.6% of cases, although it should be noted that approximately half of the cases of persistent SCI did not occur immediately after the procedure. Delayed SCI was consistently preceded by hemodynamic instability (hypotension, bleeding, tachyarrhythmia, or heart failure), and hypotension was associated with delayed SCI in 43% [20].

### 3.3. Prognostic Scores Available

The identification of risk factors for SCI could help to develop a preoperative stratification model to better categorize patients in the high-risk group. This point is essential as it could help to acutely establish the indications for the preventive use of CSFD. Only a few teams have succeeded in developing such a score, which would be of great use in daily practice. In a retrospective cohort, Mousa et al. examined the clinical data of 7889 patients treated for thoracoabdominal endovascular aneurysm repair [25]. All potential demographic and procedural variables previously associated with the occurrence of postoperative paraplegia were tracked. Most procedures were performed in elective surgery (69.6%) for aneurysmal disease (63.4%) in high-volume centers (50%). A total of 166 patients (2.1%) developed permanent SCI (transient 1.5%) with significantly worse postoperative outcomes. The authors calculated a prediction probability for SCI from their original regression (raw score) with 13 items (Table 1). The sum of the items allows the final risk score to be determined and the group to be categorized for SCI.

The authors concluded that the final multivariate logistic regression had good discrimination, a good C-statistic (AUC: 0.792, CI: 0.758–0.826), and good calibration (χ^2^ 1/4 4.9, P 1/4 0.768). In addition, prediction of SCI was consistent when patients were stratified into low, moderate, and high risk (raw scores: 0.786 and 0.738) [23].

## 4. Bundled Protocol for Spinal Cord Protection

For many years, so-called “bundled” protocols have been used in intensive care units to avoid complications related to the long-term care and management of patients with brain injury (infections, malnutrition, delirium, etc.) [27,28,29,30]. The aim is to combine several multimodal factors to improve the overall prognosis. This approach is comparable to spinal cord protection in aortic repair. The bundled SCI prevention protocol described by Scali et al. is a very good example of the favorable impact of this approach in thoracoabdominal branched or fenestrated endovascular repairs (B/FEVARs) [31]. Implementation of the protocol was associated with a significant decrease in SCI (*n* = 13 (29%) vs. 3 (2%); *p* < 0.007), especially in high-risk patients (*n* = 28 (19%) vs. 2(4%); *p* = 0.004), and improved 1-year survival (99 ± 1% vs. 90 ± 2% before the protocol; *p* = 0.05). Several reviews have demonstrated the efficacy of such strategies [32,33,34]. In our opinion, these multimodal protection methods can be divided into three different approaches and a summary of key prevention strategies for spinal cord injury is reported in Table 2.

### 4.1. Surgical Factor: Staging

Understanding the anatomy and physiology of the collateral network supplying the spinal cord has led to the strategy of the staged endovascular approach [39,40]. It is of utmost importance to give the spinal cord sufficient time to adapt its blood supply to the new condition. To this end, it has been proposed to perform aortic coverage in stages to induce expansion of the collateral network or the formation of new vessels, leading to adequate perfusion of the spinal cord [41,42,43]. The collateral network will form as long as there is sufficient flow and pressure and the pre-existing collateral perfusion is not compromised. This is particularly important if a long aortic segment is to be covered (>200 mm). Interruption of segmental perfusion of the spinal cord induces new collateral vessels in pigs within about 5 days [44,45]. The concept of ischemic preconditioning is a physiologic approach that explains the importance of a staged strategy [46]. The goal is to generate short periods of ischemia that lead to the production of various molecular mechanisms that protect the tissue or organs from a subsequent prolonged ischemic event.

Several staging strategies have been described, most notably covering the descending thoracic aorta with a large TEVAR 6 to 8 weeks before thoracoabdominal repair or delayed implantation of one of the stent-graft limbs, leaving a type Ib endoleak. A randomized, controlled trial of preoperative, minimally invasive, staged segmental arterial coil embolization is currently recruiting and may provide critical elements in favor of this strategy [47].

### 4.2. Anatomical Approach: Preservation of the Spinal Network

It is now well described that the integrity of the spinal cord may also be maintained by an extensive network of collateral vessels, including contributions from the lumbar arteries and the pelvic circulation [48]. In particular, the left subclavian artery (LSA) and the internal iliac arteries also contribute to the blood supply to the spinal cord by supplying branches that drain into the radiculomedullary vessels [8]. The mechanisms of symptomatic SCI after TEVAR have been studied in the European Registry of Endovascular Aortic Repair Complications (EuREC) [49]. Extensive coverage of intercostal arteries by TEVAR with simultaneous occlusion of at least two vascular territories supplying the spinal cord was found to be highly predictive of SCI, especially in combination with persistent intraoperative hypotension.

Risk factors for SCI include coverage of the left subclavian artery (LSA) and impairment (or sacrifice) of distal collateral inflow, including the intercostal and lumbar segmental arteries, hypogastric arteries, and sacral arteries. In this context, a history of PAD and other cardiovascular risk factors (diabetes mellitus, hypertension, renal disease, etc.) represents an important associated factor which probably explains the consequences of SCI. Therefore, every effort must be made to achieve early reperfusion of the pelvic and lower limbs and preservation or revascularization of the LSA and internal iliac artery. The contribution of the collateral network arising from the LSA and internal iliac arteries explains why patients have poorer outcomes when one or more of these branches are occluded [50].

### 4.3. Medical Approach: Optimization of Spinal Cord Oxygenation

The short duration of low spinal cord blood flow associated with SCI suggests that medical therapy with hemodynamic and metabolic interventions lasting only 24–72 h may allow routine preservation of normal spinal cord function despite the sacrifice of all vascular networks via endovascular repair of large TAAs.

- Permissive hypertension and spinal cord perfusion pressure (SCPP)

Since the irreversible damage to the spinal cord occurs 12 to 48 h after surgery, an immediate response is imperative to try to reverse spinal cord suffering and permanent impairment. Thus, increasing mean arterial pressure (MAP) appears to be the first immediate action that should be taken without further delay given the close relationship between systemic blood pressure and spinal cord perfusion [51]. In general, vasopressor agents such as noradrenaline are administered to maintain a target MAP of 80–100 mmHg and to ensure an SCPP of at least 70 mmHg [52].

SCPP = MAPd − (CSFP or CVP [whichever is greater])

SCPP: spinal cord perfusion pressure; MAPd: distal mean aortic pressure; CSFP: cerebrospinal fluid pressure; CVP: central venous pressure.
Failure to maintain a patient’s preoperative baseline arterial pressure in the early postoperative period is strongly associated with delayed postoperative SCI [53]. MAP can be further increased in 5–10 mmHg steps in case of persisting SCI.
Active CSFD can also rapidly improve PPM by lowering SCPP and must be considered in urgent cases.

- Secondary spinal cord injuries (SSCIs)

Irreversible, systemic secondary SCI is a major prognostic factor in the prevention of neurological sequelae following a lesion process. It refers to the changes that develop over a period of time (from hours to days) after the primary spinal injury. This includes a whole cascade of cellular, chemical, tissue, or vascular changes in the spine that contribute to further destruction of the spinal tissue [54]. This concept was originally developed for traumatic brain injury, but can be extended to any central neurological damage, including SCI in aortic endovascular surgery. These lesions, termed “secondary,” may be intraspinal in origin, a consequence of metabolic and inflammatory disturbances associated with the primary ischemia, or they may be systemic in origin, when failure of vital cardiorespiratory functions leads to spinal ischemia [55]. This is referred to as secondary spinal injury of systemic origin (Table 3). This concept explains the higher proportion of SCI in patients with COPD (hypoxemia) or transfusion of packed red blood cells (anemia):
  - Oxygen
  Any hypoxemia should be considered potentially dangerous. Maintaining a PaO_2_ of at least 60 mmHg (SpO_2_ > 95%) is therefore a primary goal.
  - Hemoglobin
  Evidence-based recommendations for patient blood management have recently been published [56]. A formal program must be implemented for every anemic patient prior to complex endovascular aortic repair. A hemoglobin concentration of 7–8 g/dL is the transfusion threshold that applies in intensive care units for cardiac surgery or critically ill patients. However, for patients with acute central nervous system injury, there is a lack of high-quality published data. In the absence of a clear recommendation, it is usual to consider a transfusion threshold of 10 g/dL in case of SCI [24].

### 4.4. Neuromonitoring

All efforts are now also focused on the early detection of spinal cord disorders. Time is of the essence when the spinal cord is suffering, and early diagnosis may change the patient’s functional prognosis, as irreversible damage occurs as early as 12 to 48 h postoperatively [57]. To this end, perioperative neurophysiologic monitoring methods are being developed.

- Intraoperative neuromonitoring

This technology, when available, is part of the strategy to reduce SCI real-time identification of motor or sensory neurological dysfunction so that immediate action can be taken to improve cord perfusion. In case of peroperative alteration of the motor (anterior cord) or sensory (dorsal part of the evoked potential of the spinal cord (MEP)) function, the first strategy is to increase the mean arterial pressure to reverse the alteration and return to reference values. If hemodynamic optimization is not sufficient, revascularization of the pelvis and lower limbs may be proposed if technically feasible, and finally early interruption of the procedure if possible.

- Biomarkers

For early detection of SCI, real-time biomarkers may be of utmost importance, particularly in the perioperative setting when the patient is under general anesthesia and cannot be examined clinically. Cerebrospinal fluid (CSF) appears to be the fluid of choice for this purpose, as it continuously interacts with the spinal cord tissue and is therefore predestined to reflect metabolic changes. Research about proteomic profiling from CSF has shown that these markers are released too long after the onset of acute SCI and therefore cannot be used. Studies on serum markers have also found a slow and delayed release into the bloodstream [58].
In a more recent work, changes in sensitive anaerobic metabolites with early release were studied for the first time. The authors used microdialysis of CSF during intraoperative procedures to detect severe disturbances in neurological energy metabolism in real time: lactate, lactate/pyruvate ratio, and glucose and glycerol levels. The authors reported a correlation between an increase in the lactate/pyruvate ratio, indicating the onset of anaerobic metabolism, and >50% change in motor evoked potential (MEP) in two patients. This could be a promising tool for the early detection of SCI in the future [57].

### 4.5. Pharmacologic Adjuncts

These include many pharmacological agents with antiedematous effects, but there are no prospective studies and they are mainly described in cases of open repair. The principle is to reduce the spinal cord edema caused by ischemia (steroids, mannitol), and to reduce the amounts of excitatory amino acids that play a role in spinal cord injury (naloxone) [33,45,59].

### 4.6. Basic Research on Early Detection of SCI

- Neuromonitoring

Due to the risk of false-positive results in neuromonitoring, experimental research is being conducted to improve the technique. Transesophageal MEP has been reported to provide a shorter response time in animal models [60]. Research into the possibility of stimulating and recording intercostal nerve activity has also been published [61].

- Biomarkers

The focus of current experiments on biomarkers is on structural proteins that are released into the cerebrospinal fluid and bloodstream as a result of damage to nerve tissue. However, despite the extensive literature on this topic, the diagnostic possibilities are currently limited. It has been proposed that in the future a multimodal approach be adopted to optimize spinal cord protection, combining near-infrared spectroscopy of the collateral network, neuromonitoring, and a combination of biomarkers [11].

## 5. Cerebrospinal Fluid Drainage (CSFD)

The CSFD technique was first used in experimental laboratories in the 1960s. Currently, the use of CSFD is controversial because the rate of serious complications associated with drain placement is alarmingly high and the evidence for its use in endovascular strategies is weak. Catheter-related complications are now well recognized and have probably been underestimated compared with the benefits that have been demonstrated in extensive open aortic surgery with proximal clamping. In the era of “endovascular-only” treatment, the indication probably needs to be more targeted and management ensured by experienced teams. In fact, several high-volume centers in different countries now recommend the curative use of CSFD, which is only used as a rescue measure when symptoms occur. However, early diagnosis of SCI and urgent CSF aspiration are of paramount importance and not always feasible in the absence of preoperative drainage, especially in cases of delayed symptomatology. Consequently, the preventive use of CSF extraction is nowadays a second-choice strategy and should be reserved for the most vulnerable cases.

### 5.1. Rational

Little is known about the inflammatory cascade and peri-cord edema that occur after extensive aortic coverage and spinal ischemia. The basic idea of CSF drainage is to reduce CSF pressure and thereby optimize perfusion pressure in the spinal cord by improving blood flow to the spinal cord. It is known that increasing MAP and lowering spinal CSF pressure are the most important maneuvers to reverse SCI. The most common level for placement is L4–L5.

### 5.2. Risk of CSFD and Contraindications

Several high-volume aortic centers have reported a significant dysfunction rate of 16% from a preemptive CSFD and frequent minor complications [62]. Most importantly, serious drainage complications have been consistently reported in the literature, with a rate of 3%.

The most important complication of CSF drainage is spinal hematoma, which can lead to catastrophic paraplegia. For this reason, special attention should be paid to the patient’s medication and coagulation prior to any extensive repair where there is a risk for SCI. A Delphi consensus recommends switching to aspirin for all patients on P2Y12 inhibitors or dual antiplatelet therapy. For patients on oral anticoagulation, discontinuation 2 to 5 days prior to surgery is mandatory [63], and also depends on the type of anticoagulant used and patient characteristics such as renal function.

The suggestion that up to 20% of paraplegias are due to spinal hematomas has reversed the trend in several large-volume centers to use drainage as a prophylactic strategy [64]. Particular caution should be exercised when considering prophylactic CSF drainage in patients at high risk of technical difficulties with drain insertion or in patients who have a low platelet count or high INR preoperatively. Screening for blood coagulation disorders should be carried out. In our experience, patients with degenerative diseases of the lumbar spine or under long-term curative anticoagulation are contraindicated for prophylactic CSF removal. Perioperative medication should be adapted to the recommendations in order to avoid further complications if curative CSFD is required.

### 5.3. Nantes University Protocol for CSFD Use

This alarming rate of paraplegia associated with CSFD-induced spinal cord injury has led to a shift from a preventive approach to a bail-out technique in several high-volume centers around the world. However, when changing strategies to use CSF drainage only as a rescue measure, several questions need to be evaluated: how well and quickly the patient can be neurologically assessed postoperatively, how quickly a rescue drainage can be performed by an expert, and how experienced the team is with drain placement. After all, if there is no monitoring of intraspinal pressure, any neurological signs must be recognized and treated immediately.

**Figure 4:**
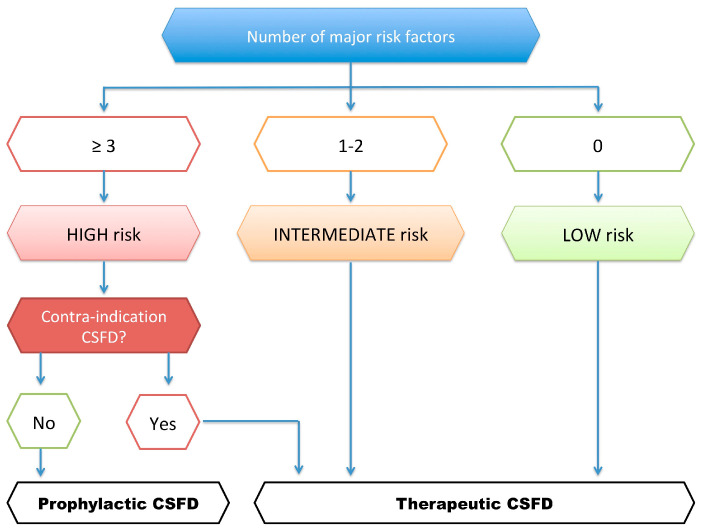
Decisional algorithm for CSFD strategy.

For this reason, we introduced a decision algorithm at our center several years ago to better define the risk of SCI and selective CSFD strategies. Taking into account the main preoperative surgical factors reported in the literature (Table 4), we can define three risk levels for the development of SCI and plan our prevention strategy based on the total number of risk factors (Figure 4) and the contraindication for CSFD. In our experience, patients with long-term anticoagulation, patients with unfavorable spinal anatomy, and patients with mental disorders have a high risk of CSFD complications and are contraindicated for preventive CSFD.

This approach may differ slightly from current recommendations. For example, the European Society of Vascular Surgery recommends placement of a CSFD with >200 mm coverage of the thoracic aorta or a previous AAA repair (class IIb, level of evidence C) [37]. In our protocol, we consider this situation as an intermediate neurological risk and refrain from prophylactic CSFD in the preoperative phase.

It is common practice to reduce or discontinue antihypertensive medications (with the exception of beta-blockers) for 48 h prior to endovascular aortic repair. We also recommend suspending antihypertensive medications for up to 30 days after the procedure.

### 5.4. Our Initial Experience after the Implementation of This Protective Protocol

We recently published our first experiences following the introduction of the protection protocol described above. From 2016 to 2021, we retrospectively analyzed our outcomes in terms of spinal cord injury (SCI rate and CFSD-related complications) in patients who underwent consecutive endovascular treatment for TAA disease. Patients were categorized as high risk (≥2 factors), intermediate risk (1 factor), or low risk (0 factors). Only high-risk patients without contraindications underwent prophylactic CSFD placement.

Among 181 patients (124 men; 69.6 years), 130 (69%) were treated for aneurysms (*n* = 24 thoracic, *n* = 28 Crawford 1-2-3, and *n* = 78 Crawford 4/pararenal); 35 (19.9%) for chronic aneurysmal dissections; and 16 (8.8%) for acute complicated type B dissections. Interventions were staged in 31 (17.2%) cases and consisted of 74 (41%) TEVARs and 107 (59%) F-BEVARs. Sixty-nine (38.1%) patients were identified as being at high risk of SCI, and sixty-four of them underwent prophylactic CFSD (four failures and one contraindication). SCI occurred in eight cases (four paraparesis cases and four paraplegias, two of which were permanent), of which three received prophylactic CSFD and five received rescue drainage. In addition, four patients developed SCI (intradural hematoma) in connection with the prophylactic CSFD, which led to one paraparesis and three paraplegias. Other CSFD-related complications were mild (6) or moderate (2), totaling 12 complications (17%). Factors associated with severe drainage-related complications were curative anticoagulation 36 h after drain removal (*n* = 1), multiple punctures (*n* = 1), platelet count < 100,000 at drain removal (*n* = 1), and bipolar disorder (*n* = 2). A total of four patients had permanent paraplegia and one had sphincter dysfunction at the last follow-up. The average follow-up time was 17 months. Mortality was 4.4% at 30 days and 13.3% at 18 months, including three (1.6%) aortic-related deaths [65].

## 6. Conclusions

Clinical research in the field of spinal cord injury prevention is struggling to keep pace with advances in surgical techniques, particularly with the advent of endovascular repair. Recent data clearly demonstrate the adverse effects and morbidity caused by drainage catheters, particularly in low-volume aortic centers. There is now an urgent need to rigorously validate the interest of lumbar drainage using pragmatic algorithms and suggest a different strategy depending on the team’s experience and the patient’s risk factors. With this considered and sensible approach, it is likely that the indications for preventive CSFD will be greatly reduced in the near future. However, this should be confirmed in future studies or large registries. Above all, the complexity not only of the procedure but also of periprocedural decision making should ensure that these procedures are only performed in high-volume centers.

## Figures and Tables

**Table 1:** Risk scoring for SCI adapted from Mousa et al. [25].

Age (by decade)	0.5
Celiac coverage	1
Current smoker	1
Dialysis	1.5
3 or more aortic devices	1
Emergent or urgent surgery	1
Adjunct procedures aorta-related	1.5
Adjunct procedures not aorta-related	1.5
Total device length 19–31 cm	1.5
Total device length ≥ 32 cm	3
ASA class 4 or class 5	1
Total procedure time > 154 min	1
High-volume center (tertiary referral center performing ≥ 50 procedures annually [26])	−1
eGFR > 60%	−1
Estimated risk for spinal cord injury	Total number of points
Low risk	0–4
Medium risk	4.5–6.5
High risk	≥7

ASA, American Society of Anesthesiologists; eGFR, estimated glomerular filtration rate.

**Table 2:** Summary of key prevention strategies for spinal cord injury in complex endovascular repair of extent 1 to 3 thoracoabdominal aneurysms.

Intervention	Rational	Potential Risks	Evidence	Class of Recommendations
Segmental artery occlusion for staged procedures	Triggers arterial collateralization and stabilizes blood supply to the spinal cord from alternate inflow sources	Extended duration of treatment, risk of aneurysm rupture, spinal cord ischemia	C	EACTS 2015: II-B
LSA revascularization	Preserves perfusion of vertebral artery	Laryngeal nerve injury, vocal cord paralysis, thoracic duct injury	Preventive	
			B–NR B C C	ACC-AHA 2022: I EACTS-ESVS 2019: II-A ESVS 2017: II-A EACTS 2015: II-A
			Curative	
			C–LD	ACC-AHA 2022: II-B
Permissive hypertension	Increases MAP to preserve blood flow in spinal cord collateral network	Cardiac dysfunction, hemorrhage	C	EACTS 2015: II-A
High hemoglobin threshold	Increases oxygen delivery to spinal cord	Transfusion-associated complications, immunization, cost	C	EACTS 2015: II-A
Perioperative neuromonitoring	Detects early impaired spinal cord function	Cost of medical devices, expertise required, false-positive rate	C C	ESVS 2017: II-B EACTS 2015: II-B
Pharmacological adjuncts	Reduce spinal cord edema or excitatory amino acids	Hyperglycemia, infection and gastrointestinal bleeding (steroids), postoperative pain (naloxone), hypovolemia (mannitol)	C	EACTS 2015: II-B
Selective CSFD drainage	Reduces CSFP and optimizes SCPP	Intracranial and neuraxial bleeding, infection, mechanical complication	Early	
			A C C C	ACC-AHA 2022: I ESVS 2017: II-A EACTS 2015: II-A ESC 2014: II-A
			Delayed	
			C C	EACTS-ESVS 2019: I EACTS 2015: II-A

ACC-AHA 2022: Circulation, 2022;146:e334–e482 [35]. EACTS–ESVS 2019: Eur J Vasc Endovasc Surg (2019) 57, 165e198 [36]. ESVS 2017: Eur J Vasc. Endovasc Surg (2017) 53, 4e52 [37]. EACTS 2015: European Journal of Cardio-Thoracic Surgery 47 (2015) 943–957 [24]. ESC 2014: European Heart Journal (2014) 35, 2873–2926 [38].

**Table 3:** Secondary spinal cord injuries.

Extra-Neurologic (Systemic)	Neurologic
Hypoxemia	Spinal cord compression (hematoma, tumor)
Low blood pressure	Vasospasm
Hypercapnia	Seizure
Anemia	Infection
Hyperthermia	
Hyperglycemia	
Hypocapnia	
Hyponatremia/Hypernatremia	

**Table 4:** Listing of major risk factors for spinal cord ischemia (adapted from Nantes university hospital protocol [64]).

Total aortic coverage > 200 mm
Coverage of the area Th9–Th12
Supra-celiac coverage > 40 mm
LSA or hypogastric coverage without immediate revascularization strategy
Prior aortic repair (abdominal and/or thoracic descending—endovascular and/or open surgery)
Prior spinal cord injury episode during TEVAR procedure or recent AAS (<15 days)
Procedure in the first 15 days following AAS

AAS, acute aortic syndrome; LSA: left subclavian artery.

## Data Availability

Not applicable.
